# The Potential Gut Microbiota-Mediated Treatment Options for Liver Cancer

**DOI:** 10.3389/fonc.2020.524205

**Published:** 2020-10-14

**Authors:** Chunye Zhang, Ming Yang, Aaron C. Ericsson

**Affiliations:** ^1^Department of Veterinary Pathobiology, University of Missouri, Columbia, MO, United States; ^2^Department of Surgery, University of Missouri, Columbia, MO, United States; ^3^University of Missouri Metagenomics Center, University of Missouri, Columbia, MO, United States

**Keywords:** liver cancer, treatments, sex, gut microbiota, clinical trials

## Abstract

Primary liver cancer is one of the leading causes of cancer death worldwide. Surgical and non-surgical treatments are optional for liver cancer therapy based on the cancer stage. Accumulating studies show that the gut–liver axis influences the progression of liver diseases, including liver inflammation, fibrosis, cirrhosis, and cancer. However, the role of gut microbiota and their derived components and metabolites in liver cancer remains to be further clarified. In this review, we discuss the roles of gut microbiota and specific bacterial species in HCC and the strategies to modulate gut microbiota to improve antitumor therapy. Given the limitation of current treatments, gut microbiota-mediated therapy is a potential option for HCC treatment, including fiber diet and vegetable diet, antimicrobials, probiotics, and pharmaceutical inhibitors. Also, gut microbiota can be used as a marker for early diagnosis of HCC. HCC occurs dependent on various environmental and genetic factors, including diet and sex. Furthermore, gut microbiota impacts the immunotherapy of HCC treatment. Therefore, a better understanding of the role of the gut–liver axis in liver cancer is critically important to improve therapeutic efficacy.

## Introduction

Liver cancer is the fourth leading cause of cancer death worldwide (1). In the United States, there will be approximately 42,030 new cases of primary liver cancer and intrahepatic bile duct cancer and 31,780 deaths due to these cancers in 2019, according to the American Cancer Society’s estimate^1^. Hepatocellular carcinoma (HCC) is the most common type of primary liver cancer (2), and the incidence of HCC is predicted to rise continually in the next decade (3). HCC typically results from chronic liver disease (4), and the main risk factors causing HCC are hepatitis B or C viruses, alcohol abuse, non-alcoholic fatty liver disease (NAFLD), diabetes, and other metabolic and genetic diseases (5, 6). Early diagnosis of HCC in patients is critically important for treatment with good outcomes (7). Unfortunately, the determination of HCC is often made in advanced disease stages, which are frequently accompanied by liver dysfunction or failure (2).

There are multiple options available for HCC treatment, including surgical resection and non-surgical therapies (8). HCC treatment options selectively depend on the stage of the disease, liver function, and cost of treatment. Even though the survival of patients with HCC is prolonged, recurrence remains a major issue for HCC treatment. In the past few years, new molecular targeting agents have been approved for systemic treatment by the United States Food and Drug Administration (FDA) (9, 10). In 2019, the FDA approved cabozantinib (Cabomeyx, Exelixis, Inc.) treatment in HCC patients as the second-line^2^. Cabozantinib is a multi-tyrosine kinase inhibitor primarily targeting tyrosine-protein kinase Met (c-MET), vascular endothelial growth factor receptor 2 (VEGFR2), and tyrosine kinase receptors AXL and RET, which was initially approved to treat medullary thyroid cancer or advanced renal cell carcinoma (RCC) (11, 12). Given the complex pathogenesis of HCC, current therapies still fail to meet the needs of patients.

Gut microbiota and gut microbiota-derived products have been shown to play important roles in the pathogenesis of HCC and its therapy. For instance, lipoteichoic acid (LTA, a Gram-negative bacterial cell wall component) and deoxycholic acid (DCA, a secondary bile acid produced by bacteria) collaboratively induced the expression of prostaglandin-endoperoxide synthase 2 or cyclooxygenase-2 (COX-2) through Toll-like receptor 2 (TLR-2) in senescent hepatic stellate cells (HSCs) to enlarge prostaglandin E_2_ (PGE_2_)-mediated inhibition of antitumor immunity, resulting in HCC progression (13). It has been reported that gut microbiota-derived products can modulate hepatic inflammation and immunity to impact non-alcoholic steatohepatitis (NASH) and virus-induced HCC progression (14). HCC patients who are responsive to anti-programmed cell death protein 1 (PD-1) immunotherapy had higher taxa richness in fecal samples compared to non-responders (15). In addition, *Akkermansia muciniphila* and *Ruminococcaceae* spp. are enriched species in responder patients, while *Proteobacteria* increased in non-responders.

In this review, we first summarize current therapies for liver cancer. Then, we discuss the potential roles of gut microbiota in liver cancer and gut microbiota-mediated treatment and diagnosis for liver cancer, specifically focusing on the shift of gut microbiota in HCC development and treatment.

## Current Therapies for Liver Cancer

Currently, there are several treatment options for liver cancer, but the selection is highly dependent on the cancer stage and remaining liver health (16, 17). Surgical resection is one of the major curative treatment options for the primary liver tumor or metastatic liver tumor (18, 19). However, surgical treatment requires to be performed in the early stage of liver cancer with a low potential incidence of metastasis. When surgical resection is not an option, minimally invasive local therapies such as radiofrequency ablation (RFA), microwave ablation (MWA), high-intensity focused ultrasound (HIFU), and irreversible electroporation (IRE) become treatable options for both primary and metastatic liver tumors (20, 21). For widespread liver cancer, chemotherapy, immunotherapy, and targeted therapy may be preferable. For example, sorafenib, a multi-kinase inhibitor with anti-proliferative and anti-angiogenic effects, has represented the primary treatment for advanced HCC for a long time (22). It was the only FDA-approved systemic therapeutic agent for HCC treatment until the recent approval of five new agents. In newly approved agents, lenvatinib is optional in the first-line treatment, while regorafenib, nivolumab, pembrolizumab, and cabozantinib are used as second-line therapies (9). All of these treatment options could be applied according to the stage and size of liver tumor. The treatment options for liver cancer are listed in Table 1.

**TABLE 1:** Current treatment options for liver cancer.

Treatments	Conditions	Examples	References
Surgical therapy	Surgical resection is an option for patients with early-stage HCC and preserved liver function. Surgical resection is commonly applied in solitary tumors ≤5 cm in size or ≤3 cm without gross vascular invasion and portal hypertension. Liver transplantation is a curative therapy for end stage liver disease.	Surgical resection, liver transplantation.	(19, 86–88)
Ablation	Ablation is a therapy to locally destroy the tumor cells with heat, rapid cooling, etc. It is applied in scattered small liver tumors. It is an effective treatment for patients with advanced primary or secondary liver tumors.	Radiofrequency ablation (RFA), microwave ablation (MWA).	(89–91)
Embolization therapy	An effective therapy for unresectable tumors by blocking or reducing the tumor blood circulation. Gene embolization selectively transfers viruses or vector embolized with cytokines (e.g., TNF-α and IFN-γ) or p53 genes.	Transarterial embolization (TAE) Transarterial chemoembolization (TACE)	(92–94)
Radiation therapy	High-energy rays or beams of intense energy are used to kill cancer cells. It can offer local treatment for unresectable HCC, but may not be a good option for some patients whose liver has been greatly damaged by diseases such as hepatitis or cirrhosis.	Photon-based intensity-modulated radiation therapy (IMRT), three-dimensional conformal radiotherapy (3D-CRT).	(95–97)
Targeted therapy	Medicines that specifically target some proteins can reach almost all parts of the body, which makes them potentially useful against cancers with metastasis. It is optional for tumors that are not very sensitive to chemotherapy.	Tyrosine kinase inhibitors: sorafenib (Nexavar) and cabozantinib (Cabometyx).	(98, 99)
Immunotherapy	Immunotherapy uses the self-immune system to fight cancer. However, cancer cells sometimes use certain checkpoints to avoid being attacked by the immune system. By blocking immune checkpoint protein PD-1, the drugs can improve the immune response against cancer cells. This treatment can shrink or slow tumor growth.	Pembrolizumab (Keytruda) and nivolumab (Opdivo).	(100–102)
Chemotherapy	Antitumor medicines to kill fast-growing cancer cells are an option for people whose liver cancer cannot be treated with surgery and is not responsive to local therapies such as ablation or embolization, or targeted therapy. Medicines for chemotherapy and targeted treatment can reach almost all parts of the body.	Oxaliplatin (Eloxatin), mitoxantrone (Novantrone).	(103, 104)

Cancer recurrence and therapeutic resistance are the main issues that reduce the survival outcomes of cancer patients (23). In this situation, combination therapy, treatment with two or more therapeutic agents or options, is helpful for good outcomes. For example, doxorubicin is a commonly used chemotherapy drug with trans-arterial chemoembolization (TACE) in HCC treatment (24). Tremelimumab, an immune checkpoint blocker, in combination with tumor ablation, is beneficial for patients with advanced HCC and viral infection as it can improve the infiltration of CD8^+^ T cells and reduce viral load (25).

## The Roles of Gut Microbiota in Liver Cancer

The liver is directly exposed to gut microbial components and metabolites via the liver portal vein (26). Increasing studies show that the gut–liver axis influences the progression of liver diseases such as liver inflammation, fibrosis, cirrhosis, and cancer (27, 28). For instance, high-alcohol-producing bacterium *Klebsiella pneumoniae* is implicated in the pathogenesis of NAFLD in human patients, evidenced by oral gavage of a clinically isolated strain causing NAFLD in mice (29). Cirrhotic patients with or without HCC had a higher abundance of genera *Lactobacillus* and *Bacteroides* with LDA scores larger than 4.0, whereas healthy controls had a higher abundance of *Akkermansia* and *Methanobrevibacter* (30). Additionally, HCC patients possessed relatively greater abundance of *Bacteroides* and *Ruminococcaceae* and lower abundance of *Bifidobacterium* compared with cirrhotic patients without HCC.

Gut microbiota impacts liver cancer by modulating different factors, including bile acids, immune checkpoint inhibitors, and Toll-like receptors (TLRs), among others.

### Bile Acids

Bile acids (BAs) consist of primary and secondary bile acids. Primary BAs such as cholic acid (CA) and chenodeoxycholic acid (CDCA) are synthesized in hepatocytes from cholesterol, while secondary BAs such as deoxycholic acid (DCA) and lithocholic acid (LCA) are synthesized by the intestinal bacteria using the primary BAs (31, 32). While BAs play pivotal roles in glucose metabolism (33) and vitamin and lipid absorption (34), an overabundance of BAs can cause hepatocyte DNA damage to promote carcinogenesis by promoting the alteration of tumor suppressor genes and oncogenes (34). Ma et al. reported that the conversion of primary to secondary BAs impacted the infiltration of hepatic natural killer T cells (NKT cells), which controlled the progression of liver cancer in mouse (35). The accumulation of hepatic CXCR6^+^ NKT cells was mediated by the expression of CXCL16 in liver sinusoidal endothelial cells (LSECs). In human samples, the presence of primary bile acid CDCA was positively correlated with CXCL16 expression, with which the expression of secondary bile acid GLCA was inversely correlated (36). The bile acid biotransformation was influenced by gut microbial community (37), such as bacterial species *Clostridium* (35). These findings indicate that modulating gut microbiota can change the components of BAs to improve antitumor immunity. Furthermore, BA receptors, farnesoid X receptor (FXR), and G protein-coupled bile acid receptor 1 (TGR5) are the potential regulators for BA homeostasis and carcinogenic effects in liver cancer (34).

### Immune Checkpoints

Immune checkpoint inhibitors are promising treatable options for HCC treatment or applied as an adjunct therapy (38). Cancer development is associated with immune suppression since cancer cells can activate different immune checkpoint pathways to inhibit antitumor therapies (39). Antibodies or inhibitors that block cytotoxic T-lymphocyte-associated antigen 4 (CTLA-4), PD-1, programmed cell death 1 ligand 1 (PD-L1), and CD24 show promising therapeutic effects on cancer treatment (39–41). Tremelimumab, a monoclonal antibody that blocks CTLA-4, was first tested in patients with HCC and hepatitis C virus infection (42, 43). The results indicated that tremelimumab treatment showed not only anti-HCC effect but also enhanced anti-HCV immunity.

Further clinical trials demonstrated the reliable adjunct antitumor effect of tremelimumab with the combination of subtotal RFA or chemoablation in patients with advanced HCC (25). The combination of anti-PD-1/PD-L1 with anti-CTLA-4 antibodies and the synergistic application of immune checkpoint inhibitors with other antitumor therapies are being evaluated at different stages of clinical trials. The results suggest that an anti-PD-1 antibody in combination with locoregional therapy or other targeted therapy is an effective treatment for HCC (44, 45). Immune checkpoint inhibitors have been shown to prolong the survival time in HCC patients (46). Therefore, Nivolumab, a monoclonal antibody that blocks the PD-1 receptor on T cells, was approved by the United States FDA for liver cancer treatment in 2017. Pembrolizumab (Keytruda), another immune checkpoint inhibitor for PD-1, was approved by the United States FDA for HCC treatment in 2018.

Importantly, increasing evidence shows that gut microbiota influences the efficacy of immune checkpoint antibodies, as antibiotic treatment can diminish their effectiveness by depletion of gut microbiome, while the presence of specific gut microbes increases this efficacy (47). Clinical studies have shown that some of the bacterial species enhanced the efficacy of immune checkpoint therapy (48), such as the effect of *Bacteroides caccae* on anti-CTLA-4 and anti-PD-1 in melanoma (49), and the impact of *A. muciniphila* on anti-PD-1 in non-small-cell lung carcinoma (NSCLC) and renal cell carcinoma (RCC) (50). Therefore, modulating gut microbial components to improve the antitumor effect of immune checkpoint inhibitors is a potential strategy for HCC treatment.

### TLRs

Toll-like receptors are the most well-studied family of pattern recognition receptors (PRRs) (51). TLRs can recognize pathogen-associated molecular patterns (PAMPs) and endogenous damage-associated molecular patterns (DAMPs) like tumor-derived antigens to activate the innate immune responses (52, 53). Gut dysbiosis, the disruption of the balance of gut microbiome, impacts the hepatic immune response through the gut-derived components like LPS and unmethylated CpG DNA, which can activate the TLR-signaling pathway (54). Even though the role of TLRs varies in different cancers (55), a series of studies have shown that targeting TLRs is a promising strategy for cancer immunotherapy (56, 57). In the liver, TLR4 and TLR9 play essential roles in the liver inflammation–fibrosis–cancer axis, as TLR4^–/–^ or TLR9^–/–^ *Tak1*Δ*Hep* mice experience reduced spontaneous HCC development compared to *Tak1*Δ*Hep* mice (58). Clinical investigations also show TLR4, the ligand of Gram-negative bacteria membrane component lipopolysaccharide (LPS) that plays a pathogenic role in chronic inflammation, a causative factor in human HCC (59). The expression of TLR9, the ligand of which is unmethylated CpG DNA in bacteria or viruses, has been positively associated with human colorectal cancer and liver metastasis (60). Thus, modulating gut microbiota to change TLR activity may serve as a therapeutic strategy for HCC therapy.

### Modulation of Gut Microbiota for Cancer Therapy

**FIGURE 1:**
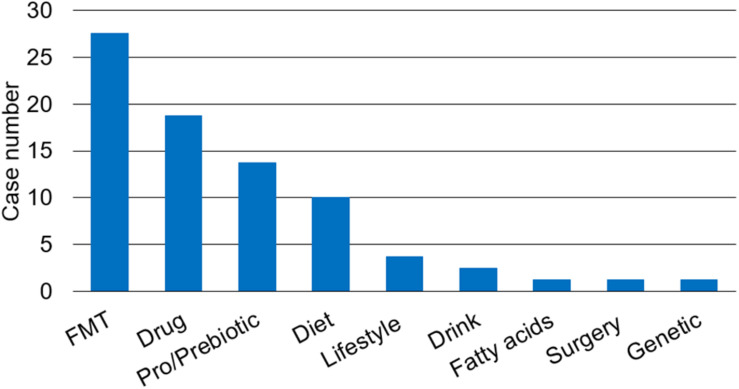
The strategies to change gut microbiota to prevent or ameliorate liver diseases in clinical trials. There are multiple strategies to restore the balance of gut microbiota such as fecal microbiota transplantation (FMT), drug therapy such as antibiotics (e.g., rifaximin) and proton pump inhibitor, pro/prebiotic, prebiotics or probiotics, change of lifestyle, and others including diet, drink, fatty acids, surgery, and genetic modification.

The composition of human gut microbiota can be modulated by various factors such as diet (61), lifestyle (62), antimicrobials (63, 64), environment (65), and diseases (66). Currently, probiotics and Fecal Microbiome Transplantation (FMT) are being investigated in cancer treatment as an adjuvant strategy to increase the efficacy of chemotherapy and immunotherapy (67). There are 80 recruiting or completed microbiota study trials associated with liver diseases on the website ClinicalTrials.gov with the keywords liver disease and microbiota, including NAFLD, NASH, fatty liver disease (FLD), alcoholic liver disease (ALD), HCC, liver encephalopathy, hepatitis, liver transplantation (LT), or resection. The strategies to affect change in the gut microbiota in those trials are summarized in Figure 1.

Overtake of soluble dietary fiber (e.g., Pectin and Fructooligosaccharide) that can be metabolized to short-chain fatty acids (SCFAs) by gut microbiota may cause cholestasis and HCC in mice, specifically with gut overgrowth of fiber-fermenting bacteria like *Clostridium* cluster XIVa (68). The authors also showed that administration of antibiotic metronidazole reduced butyrate-producing bacteria and the incidence of HCC in TLR5 knockout (KO) mice fed soluble fiber inulin-containing diet. Another study showed that vancomycin could prevent the development of HCC by selectively depleting Gram-positive bacteria *Lachnospiraceae* (Clostridium cluster XIVa), *Ruminococcaceae*, and *Bifidobacteria*, which ferment fiber and generate secondary bile acids (69). Feeding tomato powder (TP) could impede HFD plus diethylnitrosamine (DEN, injected once at 2 weeks of age)-induced HCC development in β-Carotene-15, 15′-oxygenase (BCO1), and β-carotene-9′, 10′-oxygenase (BCO2) double knockout mice (70). In addition, TP feeding altered the richness and diversity of gut microbiota, accompanying a significant decrease in the abundance of genera *Clostridium* and *Mucispirillum*. Another study reported that probiotics composed of *Lactobacillus rhamnosus* GG, viable probiotic *Escherichia coli* Nissle 1917, and heat-inactivated VSL#3 (1:1:1) could shift the gut microbiota to increase beneficial bacteria such as *Prevotella* and *Oscillibacter*, resulting in a reduction of HCC growth and Th17 cell differentiation (71). VSL#3 contains *Streptococcus thermophilus*, *Bifidobacterium breve*, *Bifidobacterium longum*, *Bifidobacterium infantis*, *Lactobacillus acidophilus*, *Lactobacillus plantarum*, *Lactobacillus paracasei*, and *Lactobacillus delbrueckii* subsp. Combined (synbiotic) prebiotic *B. infantis* and probiotic milk oligosaccharide treatment reverses Western diet (WD)-induced NASH in FXR knockout mice (72). Moreover, bariatric surgery, such as Roux-en-Y gastric bypass and laparoscopic sleeve gastrectomy, can induce the shift of gut microbiota to reduce obesity and weight loss (73), showing a promise in NAFLD and NASH (74). Thus, it may be a potent treatment option for early stage of NASH-HCC patients.

### Gut Microbiota as a Non-invasive Biomarker for HCC

**FIGURE 2:**
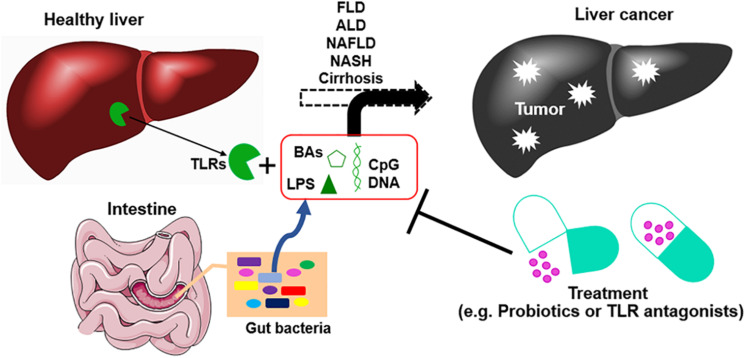
The development of liver cancer and gut microbiota-mediated therapy. Chronic liver diseases including viral infections, fatty liver disease (FLD), alcoholic liver disease (ALD), non-alcoholic fatty liver disease (NAFLD), non-alcoholic steatohepatitis (NASH), and cirrhosis without effective treatments can lead to liver cancer. Dysbiosis of gut microbiota promotes the progression of this process by leakage of gut microbial products such as deoxycholic acid (DCA), lipopolysaccharide (LPS), and unmethylated CpG DNA. These bacterial products promote liver inflammation, fibrosis, and cirrhosis. Modulation of gut microbiota by applying probiotics, prebiotics, and antibiotics, or using antagonists of bacterial products, can improve gut barrier and reduce the progression of the liver inflammation–fibrosis–cirrhosis–cancer axis. BAs, bile acids; TLR, Toll-like receptor.

Early diagnosis of HCC comes with multiple treatment options and typically leads to good outcomes. Biomarkers including Alpha-fetoprotein (AFP), Lens culinaris agglutinin A-reactive fraction of alpha-fetoprotein (AFP-L3), and des-gamma-carboxy prothrombin (DCP) have been established as HCC-specific tumor markers (75, 76). New potential biomarkers, such as Aldo-keto reductase family 1 member 10 (AKR1B10) (77), are being investigated for the diagnosis and prognosis of HCC. Changes in the gut microbiome may also serve as biomarkers of disease as they have been associated with the progression of liver diseases, from fibrosis/cirrhosis to cancer (78, 79). For example, the abundance of fecal *Enterobacteriaceae* and *Streptococcus* is increased in patients with cirrhosis, while the abundance of *Akkermansia* is reduced. In HCC patients, *Bacteroides* and *Ruminococcaceae* were increased, while *Bifidobacterium* was reduced. Further study showed that *Akkermansia* and *Bifidobacterium* were inversely correlated with inflammatory marker calprotectin (30). These results indicated that during the development of HCC, a group of bacteria are associated with different stages of disease and tumor progression. A better understanding of the association of gut microbiota with liver cancer leads to a therapy option. Potent gut microbiota-mediated liver cancer therapies are summarized in Figure 2.

## Discussion

Liver cancer is a leading cause of cancer deaths worldwide. Liver resection or transplantation is the curative treatment for HCC, but late diagnosis and lack of donor organs reduce the survival rate. Given these limitations, many non-surgical treatment options are available for advanced stages of HCC. However, the cost for some current treatments like sorafenib is relatively high, which may be associated with adverse or variable effects (80). Modulating gut microbiome is a potential option for liver cancer treatment and diagnosis. HCC occurs about three times more in men than in women (81). Therefore, sex is also another consideration when choosing gut microbiota-mediated treatment. In a streptozotocin–high-fat diet (STZ-HFD)-induced NASH-HCC murine model, male mice possessed a higher abundance of some specific genera than female mice, including *Clostridium*, *Corynebacterium*, *Bacillus*, *Desulfovibrio*, and *Rhodococcus*, which were associated with higher HCC incidence (82). Data from prospective cohort studies indicate that intake of vegetables reduces the risk of liver cancer development, especially for men (83). LT can also alter gut microbial profile. The abundance of bacteria, such as *Actinobacillus*, *Escherichia*, and *Shigella*, decreased post-LT compared to pre-LT, whereas the abundance of bacteria, such as *Micromonosporaceae*, *Desulfobacterales*, the *Sarcina* genus of *Eubacteriaceae*, and *Akkermansia* increased (84). Furthermore, features of the gut microbiota are also associated with hepatitis virus- and non-hepatitis virus-related HCC, evidenced by the fact that hepatitis B-HCC patients harbor much more pro-inflammatory bacteria such as *Escherichia*/*Shigella* and *Enterococcus*, but less amount of *Faecalibacterium*, *Ruminococcus*, and *Ruminoclostridium* relative to healthy controls (85). Therefore, precise analysis of the change of gut microbiota of each individual in the development of HCC is critically essential for modified treatment. Those recent findings suggest that microbiome-mediated therapeutic options can be applied to treat liver cancer as well as the early stage of chronic liver diseases, which may conquer the drawbacks of current therapies, such as the presence of metastasis and liver dysfunction. However, more clinical trials evaluating gut microbiota-mediated therapies are necessary to improve outcomes of HCC treatment.

## Author Contributions

CZ and MY conceived and wrote the manuscript. AE critically reviewed and revised the manuscript. All authors contributed to the article and approved the submitted version.

## Conflict of Interest

The authors declare that the research was conducted in the absence of any commercial or financial relationships that could be construed as a potential conflict of interest.

## Footnotes

1 https://www.cancer.org/cancer/liver-cancer/about/what-is-key-statistics.html

2 https://www.fda.gov/drugs/fda-approves-cabozantinib-hepatocellular-carcinoma
